# Endodontic Perforation Closure by Five Mineral Oxides Silicate-Based Cement with/without Collagen Sponge Matrix

**DOI:** 10.1155/2021/4683689

**Published:** 2021-09-07

**Authors:** Talal Al-Nahlawi, Maisour Ala Rachi, Amjad Abu Hasna

**Affiliations:** ^1^Operative Dentistry and Endodontics Department, Syrian Private University (S.P.U), Damascus, Syria; ^2^Department of Restorative Dentistry, Endodontics Division, Institute of Science and Technology, Sa˜o Paulo State University – UNESP, Sa˜o José Dos Campos, São Paulo, Brazil

## Abstract

Endodontic perforations are common accidents that occasionally happen as a result of misuse or difficult anatomy of some teeth; it may lead to teeth loss unless a good management is provided. Bioceramic (silicate-based) cements like mineral trioxide aggregate have a big role in management of such accidents. This case report aimed to evaluate the ability of five mineral oxides cement “5MO” in sealing two root canal perforations (furcation and postdrill perforations) and inducing clinical and radiographic healing in the periodontal tissues with/without the use of collagen sponge matrix. A 58-year-old healthy female was referred to our dental office complaining of severe pain in the upper left premolars' region. Periapical radiographic examination revealed unsatisfactory root canal treatment of the teeth #24 and #25 with a furcation perforation and a postdrill perforation, respectively. Cone-beam computed tomography “CBCT” scans confirmed the findings of the periapical radiography and revealed the presence of radiolucent lesions surrounding the apex of both teeth #24 and #25. The treatment plan was a nonsurgical root canal retreatment by endodontic access through the full-ceramic crowns. After three years of follow-up, CBCT scans revealed a complete healing and bone formation on both premolars. This case report indicates the use of 5MO cement for endodontic perforations management.

## 1. Introduction

Endodontic perforation is defined as the communication of the root canal system with the periodontium, it is considered as an accident of the root canal treatment “RCT,” and it may lead to tooth loss unless a good management is provided [1, 2]. Conversely, it results from noniatrogenic factors like progressive internal resorption or active caries lesion [3].

Regeneration in endodontics started in the last century, with some attempts by researchers, to induce regeneration and repair of exposed cavities [4, 5]. Calcium hydroxide “Ca(OH)_2_,” beside others, was the material of choice for tissue regeneration and repair [6], until the mineral trioxide aggregates “MTA” release at the end of the twentieth century as the first biocompatible and reparative silicate-based cement [7]. MTA is an effective material in sealing of lateral and furcation perforations [7, 8], and it can be applied using both immediate and mediate techniques [9]. Furthermore, it is indicated for apexogenesis, apexification, and revascularization of vital and nonvital immature teeth [10–12]. Later, several bioceramic (silicate-based) cements of the same chemical formula were released [13, 14].

Five mineral oxides “5MO” is a new repair silicate-based cement, derived from Portland cement, developed to treat all endodontic complications and accidents (patent). As a capping material, it is effective as Ca(OH)_2_ (Dycal) [15] and MTA [16] and as a root-end filling material in apicoectomy [17, 18].

Collagen sponge is a biodegradable material, indicated as a surgical tampon [19]. In some endodontic perforations, it may be indicated as a matrix for the repair cement insertion [20] as it does not interfere in the healing process [21].

The aim of this case report was to evaluate the ability of 5MO in sealing two root canal perforations (furcation and postdrill perforations) and inducing clinical and radiographic healing in the periodontal tissues with/without the use of collagen sponge.

## 2. Case Report

### 2.1. Case History

A 58-year-old healthy female was referred to our dental office complaining of severe pain in the upper left premolars' region with no history of trauma. However, the patient related a previous root canal treatment of most of her upper teeth, including the premolars, as a demand for “Hollywood smile,” according to the indication of the anterior professionals in the past six months, even without a pathological indication.

### 2.2. Patient Assessment

The clinical investigation revealed severe pain (positive response) to vertical percussion and digital palpation in the periapical region of tooth #24. However, the neighboring teeth #23 and #25 presented negative responses for the same exams. There was no fistula nor swelling related to the evaluated teeth. The crowns were well-adapted without any infiltration, and the periodontal exploration revealed gingival pocket varied between 1 and 3 mm with various exploring locations and grade I mobility. Teeth #23–25 were tested by the pulp vitality test (the cold test) as detailed in a previous study [22]. Teeth #24 and #25 presented negative responses; conversely, tooth #23 was vital with characteristics of healthy pulp.

Periapical radiographic examination revealed unsatisfactory root canal treatment of the tooth #24 presenting: (I) missed lingual canal; (II) furcation perforation (obturated and condensed with gutta-percha); (III) susceptible broken instrument in the furcation area; and (IV) broken instrument in the apical third of buccal canal. The remaining part of the buccal canal was obturated with gutta-percha, and a fiber post was inserted in the coronal part; then, the tooth was crowned.

**Figure 1:**
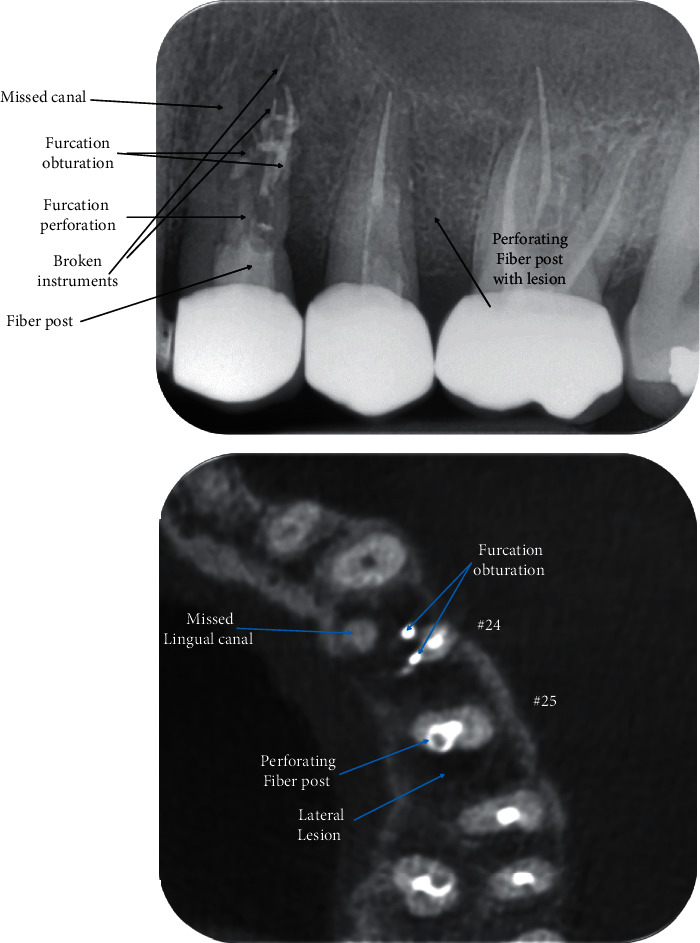
The initial periapical radiography and the axial section of CBCT scans of the teeth #24 and #25.

In addition to an unsatisfactory root canal treatment of the tooth #25 is associated with a lateral lesion because of postdrill perforation. Cone-beam computed tomography “CBCT” scans confirmed the findings of the periapical radiography and revealed the presence of radiolucent lesions surrounding the apex of both teeth #24 and #25 (Figure 1).

The final diagnosis was acute periapical periodontitis of tooth #24 and chronic periapical periodontitis of tooth #25. The treatment plan was a nonsurgical root canal retreatment by endodontic access through the full-ceramic crowns with final composite resin restoration for teeth #24 and #25. An informed consent form was signed by the patient agreeing to undergo the treatment plan and authorizing the publication of the case report.

### 2.3. Clinical Interventions

#### 2.3.1. Tooth #24

**Figure 2:**
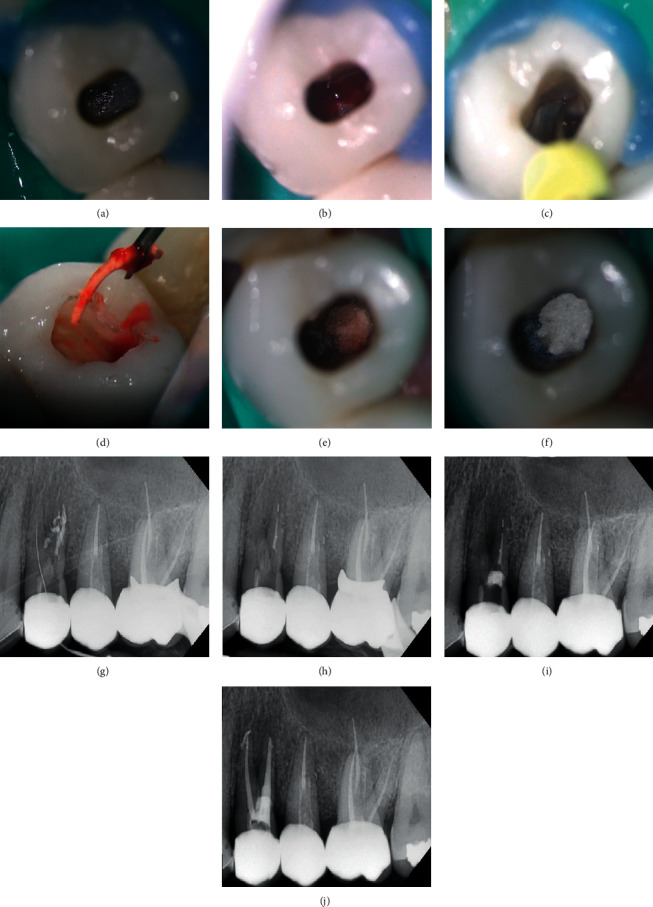
Clinical images and periapical radiographs of the retreatment of tooth #24. (a) Access cavity; (b) bleeding perforation; (c) exploring the palatal canal; (d) gutta-percha removal from the perforation; (e) collagen sponge placement; (f) 5MO cement application; (g) radiography of palatal canal localization; (h) cleaned perforation site; (i) radiography of 5MO cement application; and (j) radiography of obturation.

After rubber dam isolation and under microscopic magnification by SmartOptic dental microscope (Diplomat Dental, Piestany Slovakia), the access cavity was performed through the full-ceramic crown using a straight fissure diamond bur until 5 mm of depth and before reaching the furcation area (Figure 2(a)). Then, Start-X #5 ultrasonic tip (Dentsply Sirona, São Paulo, SP, Brazil) was used to remove the composite resin restoration covering the furcation perforation using the P5 NEWTRON ultrasound activator (Acteon, Indaiatuba, SP, Brazil) at 6-frequency (Endo function). Once the perforation was uncovered, a considerable amount of bleeding came out (Figure 2(b)).

After bleeding control and perforation site evaluation (3 × 3 mm), the lingual canal was detected and scouted using K-file #10 (Dentsply, Petropolis, RJ, Brazil) (Figure 2(c)). Periapical radiography confirmed the detection of the lingual canal (Figure 2(g)). Disinfection of the perforation site was done using sodium hypochlorite “NaOCl” 1%. The site was dried by cotton pellet, and Ca(OH)_2_ powder (Sultan HealthCare, York, PA, USA) was mixed with sterile saline solution and applied over the perforation site. The tooth was restored by temporary filing of Coltosol (Coltene, Rio de Janeiro, RJ, Brazil) and sealed by a layer of glass ionomer cement (Ivoclar Vivadent Ltda, Barueri, SP, Brazil) for 14 days.

In the second session, the pain was completely controlled. The temporary filling was removed, and the Ca(OH)_2_ dressing was washed away using sterile saline solution irrigation; then, the Zumax micro tweezer (Zumax medical, Suzhou New District, China) was used to catch and remove the obturation material (gutta-percha) from the perforation site (Figure 2(d)). Then, ultrasonic irrigation was performed using U-file (NSK, Suzano, SP, Brazil) at 2-frequency (Endo function) and sterile saline solution to remove any remaining materials from the furcation perforation including the susceptible broken file inside the furcation perforation.

Periapical radiography confirmed the removal of all foreign bodies from furcation perforation (Figure 2(h)). Then, a collagen sponge was packed inside the furcation perforation to form a matrix (Figure 2(e)), and the 5MO silicate-based cement (Golden Yatti LLC Muscat, Oman) was applied using the microapical placement system “MAP” (Dentsply Tulsa, Switzerland) over the collagen matrix to seal the large perforation site (Figure 2(f)). A wet cotton pellet was applied over the 5MO to accelerate the complete setting of the material, paying attention to keep the lingual canal entrance free. Last, the 5MO was sealed with glass ionomer cement (Ivoclar Vivadent Ltda, Barueri, SP, Brazil).

Then, the fiber post in the buccal canal was removed by penetrating and enlarging the fiber post from inside using LN bur size 0.6 (Dentsply Maillefre, São Paulo, SP, Brazil) and Pesso reamers files sizes 1–3 (Dentsply Maillefre, São Paulo, SP, Brazil); then, the remaining part of the fiber post was drilled away using ultrasonic tip ET20 (Acteon, Indaiatuba, SP, Brazil) till the pink color of the gutta-percha appeared.

The obturating material was removed using D3 retreatment rotary file (Dentsply Maillefer, São Paulo, SP, Brazil) at a 300 rpm rotation speed associated with NaOCl 5.25% irrigation (Figure 2(i)). The apical broken file was bypassed using manual K-files sizes #6, 8, and 10. Then, buccal and lingual canals were instrumented with rotary files Revo S rotary systems 25.06 (MicroMega, Besancon, France). Ultrasonic irrigation was performed using U-file at 2-frequency (Endo function) and NaOCl 5.25%. Then, the canals were dried with paper points 25.06 (Dentsply Maillefer, São Paulo, SP, Brazil) and obturated using the single cone technique with a 25.06 gutta-percha cone (Dentsply Maillefer, São Paulo, SP, Brazil) and bioceramic sealer (CeraSeal, Meta Biomed, Colmar, PA, USA) (Figure 2(j)).

#### 2.3.2. Tooth #25

**Figure 3:**
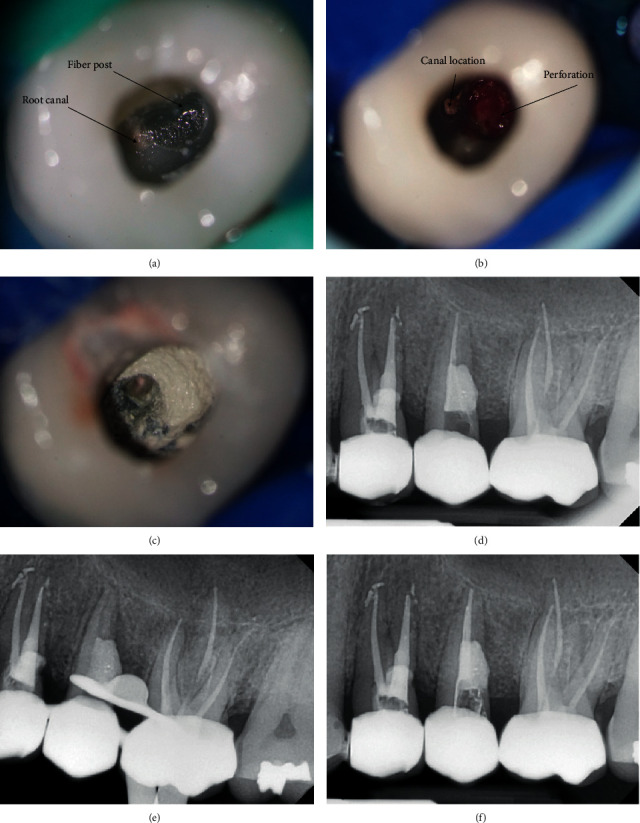
Clinical images and periapical radiographs of the retreatment of tooth #25. (a) Access cavity; (b) bleeding perforation; (c) 5MO cement application; (d) radiography of 5MO cement application; (e) radiography of cleaned and prepared canal; and (f) radiography of obturation.

Again, the same steps were followed, until reaching the fiber post (Figure 3(a)); however, while removing it with the ultrasonic tip ET20, the fiber post was pushed out into the drill post (lateral) perforation because the fiber postpreparation extends out of the root canal in a distal direction (Figure 3(d)). Later, the fiber post was removed (Figure 3(b)) using a long and narrow endo probe with the aid of ultrasonic tip ET25 (Acteon, Indaiatuba, SP, Brazil) associated with copious irrigation of the perforation site. Ca(OH)_2_ dressing was also applied over the perforation site till the next session.

The perforation site, measuring around 3 × 3 mm, was sealed using 5MO silicate-based cement (Figure 3(c)), without a collagen sponge application. The cement did not overextrude, and this was confirmed radiographically (Figure 3(e)). The same steps were followed again for retreating and obturating the single root canal of tooth #25 (Figure 3(f)). Both teeth were restored with composite resin restoration (3M ESPE, Seefeld, Germany) after a base of glass ionomer cement.

### 2.4. Follow-Up and Outcomes

One month later, the patient had no postoperative complications, and an intraoral evaluation was performed to investigate any swelling, edema, or fistula. The patient related comfort and satisfaction with the treatment.

**Figure 4:**
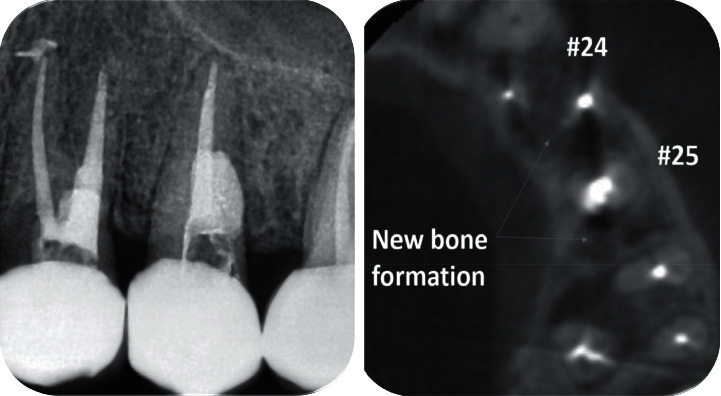
Periapical radiography and CBCT image after a 3-year follow-up.

Three years later, another follow-up session was performed. The clinical intraoral examination revealed a well-adapted full-ceramic crown, without signs of caries or marginal infiltration. Radiographically, healed periapical and lateral lesions were noticed, and a bone neoformation was observed (Figure 4(a)). Last, the CBCT scans presented complete healing and bone formation on both premolars. The patient was advised to retreat the upper adjacent molar as a radiolucent lesion was detected (Figure 4(b)).

## 3. Discussion

The adequate treatment planning demands an accurate diagnosis [23]; for this, CBCT scans were used in this case report due to its high resolution and its effectivity in determining the relationship between the root apexes and the adjacent anatomical structures [24], beside its capacity to localize the perforations' sites. In this case report, both furcation and postdrill perforations were closed by 5MO silicate-based cement using the mediate technique because of the presence of lesions associated to these perforations. The proposal was that Ca(OH)_2_ dressing will disinfect, dry the exudate, and elevate the pH at the perforation site before sealing with bioactive material [25]. MTA has presented good results in both immediate and mediate techniques [9]. Therefore, 5MO silicate-based cement should be evaluated in both techniques.

The silicate-based cement sealing should be associated with root canal disinfection to guarantee the success of the root canal treatment in perforated [26] or immature teeth [18]. MTA presented a satisfactory antibacterial effect over some facultative bacteria, but no effect over strict anaerobic bacteria [27]. This antimicrobial action is explained by the alkaline pH of bioceramic or silicate-based cements that tends to increase over time [28]. However, the 5MO antimicrobial action was not studied yet. Therefore, it indicated the use of antimicrobial agents in this study.

It was stated in the literature that the adequate disinfection of the root canal system and the sealing site, associated with the sealing ability of 5MO bioceramic cement results in satisfactory clinical and radiographic healing [18]. This disinfection was achieved in the current case report by the antimicrobial action of sodium hypochlorite as endodontic irrigant [29, 30] and calcium hydroxide as a dressing [31]. Ultrasonic activation was used to improve the endodontic irrigant penetration [30].

Other factors play a major role in healing, and preventing the lesion progression is the matrix metalloproteinases deactivation and endotoxins (LPS and LTA) detoxification, and this may be achieved by the irrigation of the root canal system by sodium hypochlorite [30, 32], besides the sealing ability of the 5MO cement [18] and the good biocompatibility, osteoconductivity, and osteoinductivity of silicate-based cements [33].

In this case report, the furcation perforation was managed using collagen sponge to serve as a matrix to prevent 5MO silicate-based cement extrusion [34]. Conversely, in the postdrill perforation, there was no need to use a collagen sponge as the perforation was lateral, and the condensation forces were applied gently with large cut paper point in apical direction, in addition to the presence of inflammatory lateral tissues that served as a matrix [35].

5MO was the material of choice in this case report because of its sealing ability stated previously [15, 17, 18]; therefore, 5MO can be used as an effective silicate-based cement in perforations management; however, more case reports and clinical studies should be realized to evaluate its cytotoxicity and tissue repair ability for long periods.

## 4. Conclusion

It was concluded that 5MO is considered as effective silicate-based cement in furcation and postdrill perforations closure with and without a collagen matrix.

## Data Availability

No data were used to support the findings of this study.
